# LEARNING CURVE IN PONSETI METHOD – EVOLUTION IN 5 YEAR-INTERVALS

**DOI:** 10.1590/1413-785220243201e273739

**Published:** 2024-03-22

**Authors:** Tatiana de Moura Guerschman, Monica Paschoal Nogueira

**Affiliations:** 1 Instituto de Assistência Médica ao Servidor Público Estadual de São Paulo (IAMSPE), Post-Graduation in Health Sciences, São Paulo, SP, Brazil.; 2 Hospital do Servidor Público Estadual de São Paulo (HSPE), Department of Orthopedics, São Paulo, SP, Brazil.

**Keywords:** Learning Curve, Clubfoot, Education, Medical, Inservice Training, Curva de Aprendizado, Pé Torto Equinovaro, Educação Médica, Capacitação em Serviço

## Abstract

**Objective:**

Evaluate whether the experience of the surgeon could reduce Ponseti treatment time, and a number of cast changes, and the evolution of the Pirani Score.

**Methods:**

2 reference centers were evaluated. At Institution 1, 254 patients with idiopathic clubfoot (403 feet) were included, and at Institution 2, 32 patients (51 feet). At institution 1 (mentor), 3 intervals of 5 years each were analyzed. At the Institution 2 (trainee), 1 interval of 5 years was analyzed.

**Results:**

Patients treated by the mentor had fewer casts compared with the trainee (p < 0.001). At Institution 1, the three mentor intervals showed differences in the number of casts (p < 0.05). A statistically significant difference was observed only in the first mentor interval (2000 to 2005, average of 3.47 casts) compared with the 2 other intervals (2005 to 2010; average of 2.6 casts and 2011 to 2015; average of 2.79 casts; p < 0.0001). Pirani score decreases the most until the third clinic visit.

**Conclusion:**

The mentor’s greater expertise was associated with fewer casts and shorter time to obtain correction in isolated clubfoot, especially right after the first 5 years of practice. Progression of the Pirani score in both institutions occurs between the first and the third casts. ***Level of Evidence III; Therapeutic Study, Retrospective Comparative Study***.

## RESUMO

**Objetivo:**

Avaliar se a experiência no Método Ponseti pode reduzir o tempo de tratamento e o número de gessos.

**Métodos:**

Na instituição 1 foram incluídos 254 pacientes com pé torto idiopático (403 pés) e na instituição 2, 32 pacientes (51 pés). Na instituição 1 (mentora) foram analisados 3 intervalos de 5 anos. Na instituição 2 (estagiária), foi analisado 1 intervalo de 5 anos.

**Resultados:**

Os pacientes tratados pelo mentor tiveram menos gessos em comparação aos tratados pelo estagiário (p < 0,001). Na Instituição 1, os três intervalos de mentores apresentaram diferenças no número de gessos até a correção dos pés (p < 0,05). Diferença estatisticamente significativa foi observada no primeiro intervalo do mentor (2000 a 2005, média 3,47 gessos) em comparação com os outros 2 intervalos (2005 a 2010; média 2,6 gessos e 2011 a 2015; média 2,79 gessos; p < 0,0001). O escore de Pirani diminui mais até a terceira consulta clínica.

**Conclusão:**

A maior expertise do mentor no Método Ponseti esteve associada ao menor número de gessos e ao menor tempo para correção do pé torto, principalmente logo após os primeiros 5 anos. A maior progressão do score de Pirani ocorre entre o primeiro e o terceiro gesso. ***Nível de Evidência III; Estudo Terapêutico, Estudo Comparativo Retrospectivo***.

## INTRODUCTION

Idiopathic clubfoot is one of the most common birth defects occurring in one in 1000 live births.^1,2^ Natural evolution of the deformity has a major impact on family’s social and emotional issues.^3–5^ Until the beginning of the XXI century, treatment of clubfoot was essentially surgical, but results were poor and complications, including stiffness of the ankle and subtalar joint, pain, arthritis, residual deformity, and muscle weakness, were high.^1^ Ponseti Method revolutionized the history of clubfoot. Difusion of Ponseti Method was increased by the publication of Dr Ponseti 30 year results in JBJS, in 1995, and his book in 1996^2,3^. Orthopaedic services globally started to learn and apply Ponseti Method around 2000’s and international publications reflect this tendency.^2^ Compared with the traditional 2 day course/symposium format, the mentorship educational model can reduce complications and increase the effectiveness. However, transition to this new technology was not simple.^6^ The usual structure for orthopedic surgeon’s training needed to be revised. In 1999, Shafique Pirani introduced a fast and reproducible score.^7,8^

With diffusion of Ponseti Method, without adequate training, the number of complications increased. Medical training through mentorship educational model was able to reduce complications and improve effectiveness and efficiency of the Ponseti Method.^4,9^ In medical literature there are no specific studies on the learning curve in the Ponseti Method. The purpose of this retrospective cohort study was to demonstrate the orthopedic surgeon’s learning curve in the Ponseti Method to treat idiopathic clubfoot considering the number of casts, treatment time and the correction progression according to the Pirani Score. Primary questions were: (1) Does greater experience in the Ponseti Method reduce the number of casts and treatment time? (2) Is the evolution of clubfoot correction through the Pirani Score modulated by the expertise in the Ponseti Method?

## METHODS

This is a retrospective comparative cohort study. Data from 2 reference services with orthopaedic surgeons trained in Ponseti method were evaluated. At institution 1, patients undergone treatment by a senior orthopedic surgeon with more years in practice. In this center, 254 patients diagnosed with idiopathic clubfoot (403 feet) met the eligibility criteria. In institution 2, where patients were treated by an orthopedic surgeon with less years of practice, 32 patients with idiopathic clubfoot were included (51 feet).

In institution 1, data obtained from medical records were analyzed at 3 intervals of 5 years each, considering the total time of Ponseti Method practice (2000 to 2005, 2005 to 2010 and 2011 to 2015) totaling a period of 15 years. In the Institution 2, data were analyzed in 1 interval of 5 years (2011 to 2015). In both institutions, treatment was carried out with strict adherence to Ponseti Method.

Just after residency, the orthopaedic surgeon from institution 2 (TMG - *trainee*) and the orthopaedic surgeon from institution 1 (MPN - *mentor*) developed an academic relationship also based in mentorship to refine the practice in the Ponseti Method.

The learning curve in the Ponseti method was characterized by the average number of casts in each period of the mentor and the trainee.

Patients started treatment at 14 to 180 days of age. The minimum follow up was 15 weeks after Achilles tenotomy (last event to be included in data analysis). Cases without tenotomy were not included in the study. Neurological and or syndromic clubfoot, and with any previous surgery were excluded.

Two hundred and eight-six patients were evaluated, in a total of 454 feet. At Institution 1, the mentor treated 403 feet (88,76%) and data were analyzed at 3 intervals: from 2000 to 2005, from 2005 to 2010 and from 2011 to 2015. At Institution 2, the trainee treated 51 feet (11.24%) and data were analyzed in one interval from 2011 to 2015. Patients were consecutive, and not randomized in this study. Institution 1 is a large tertiary hospital, with residency in orthopedics and fellowship in Pediatric Orthopedic Surgery, serving a metropolitan area with an estimated city population of 12 million people in 2020. Care at institution 1 was managed only by the orthopedic surgeon (mentor) who has been practicing there for 15 years and is the head of the Pediatric Orthopedic Department.

Institution 2 consisted of a small secondary pediatric hospital, with an estimated city population of 162,000 people in 2020. At institution 2 care was managed only by the orthopedic surgeon (trainee), who had 2 years of practice after residency in Pediatric Orthopedics. Quantitative variables were: age (in months), time until tenotomy (in days) and number of casts. Qualitative variables were: gender, side, and professional responsible for the patient (mentor or trainee).

We compared the quantitative variables. Time until tenotomy was shorter in patients treated by the mentor (p < 0.001), the mentor needed fewer casts to obtain correction (p < 0.001), the age of patients treated by the mentor and the trainee did not have statistical relevance (p = 0.0973).

The analysis of the quantitative variables age (in months), time until tenotomy (in days) and number of casts were done with the t-Student Test. For all tests the significance level was 5%. Another quantitative variable was the correction evolution trough the Pirani Score. Comparison of the number of casts in the different time intervals was done by the ANOVA variable analysis.^10^ Averages of the Pirani Score, number of casts and time until tenotomy were compared between the sides right and left. In this analysis, only patients with bilateral clubfoot were compared. As the different sides were from the same patient, the statistical analyses used was the Generalized estimating equation (GEE).^11–13^ Model was adjusted considering normal distribution and unstructured correlation structure (significance level was 5%).

Correlation between the severity of the foot according to the Pirani Score and time to tenotomy was evaluated according to the Spearman coefficient (closer to +1 and -1, stronger the correlation). Evolution of the Pirani score through the appointments was analyzed, as well as this correlation and its comparison between genders and laterality through the approach of mixed models with repeated measures. For all comparisons was considered 5% as significance level.

### Code availability

The authors confirm that the data supporting the findings of this study are available within the article [and/or] its supplementary materials. The data sets used and/or analysed during the current study are available from the corresponding author on reasonable request. The data are not publicly available due to information that could compromise the privacy of research participants.

### Ethics approval

This study was approved by Institutional Ethics Committee under opinion number 1.365.728.

### Consent to participate

Written informed consent according to the Declaration of Helsinki was obtained from all study participants and in applicable cases their parents or legal guardians.

### Consent for publication

Written informed consent was obtained from all patient/parents/legal guardians for publication of this study and any accompanying images and videos. A copy of the written consent is available for review by the Editor of this journal.

## RESULTS

Patients treated by the mentor needed fewer casts to obtain correction (average of 2.8 cats) than patients treated by the trainee (average of 5.5 cast; p < 0.01). Time until tenotomy was shorter in patients treated by the mentor, average of 15.9 days for patients treated by the mentor and 42.7 days, patients treated by the trainee (p < 0.01). (Table 1)

**Table 1:** Comparison of professionals.

		Professional	
		**1**	**2**	**p-value**
Time until tenotomy	n	371	43	< 0.001
	Average	15.9	42.7	
	Median	14.0	32.0	
	Stardard deviation	10.0	34.2	
	Minimum value	2.0	7.0	
	Maximum value	70.0	158.0	
Number of casts	n	403	51	< 0.001
	Average	2.8	5.5	
	Median	3.0	4.0	
	Stardard deviation	1.3	3.3	
	Minimum value	1.0	1.0	
	Maximum value	7.0	14.0	

**Figure 1:**
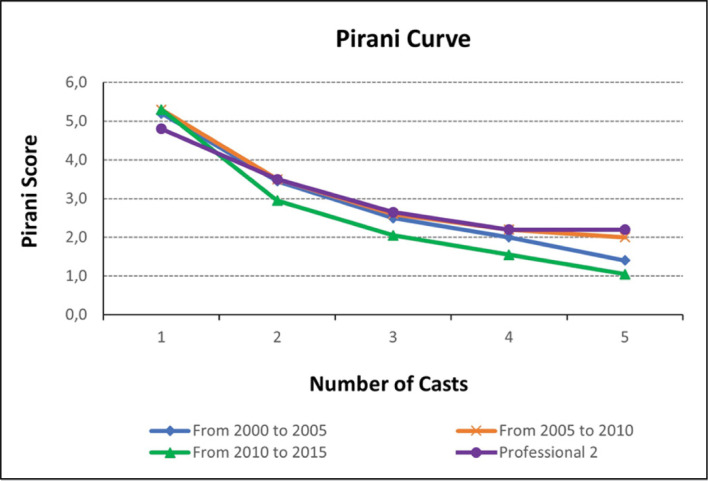
Pirani curve at all intervals (consecutive scores for every visit).

Regardless the professional, the Pirani score decreases the most until the third clinic visit, without differing in the subsequent appointments. (Figure 1)

There was no correlation between severity of the Pirani score and time until tenotomy (Spearman correlation 0.318).

There was a significant difference in the number of casts until correction between the 3 analyzed mentor intervals (p < 0.05).

**Figure 2:**
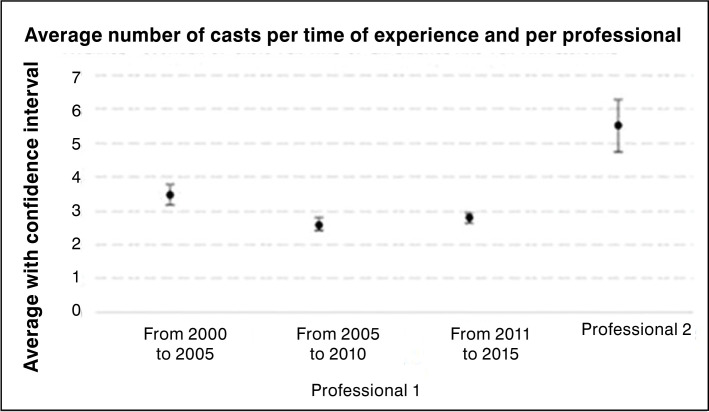
Average number of casts per length of experience and per professional.

Only the first interval (2000 to 2005) compared to the others had statistical difference (p < 0.05), evidencing that after 5 years of experience the mentor needed fewer casts to obtain correction. In the first mentor interval (2000 to 2005) the average number of casts per patient until correction of the deformities was 3.47. In the second interval (2005 to 2010) was 2.6 and in the third interval the mentor needed an average of 2.79 casts to obtain correction, featuring a plateau. The mentor evolution shows that the number of casts per patient decreased as the experience increased over time. (Table 2, Figure 2)

**Table 2:** Comparison between different periods of professional 1

		Professional 1			
		From 2000 to 2005	From 2005 to 2010	From 2011 to 2015	Professional 2
Number of patients		39	96	118	33
Number of Casts	Number of feet	53	149	201	51
	Average	3.47	2.6	2.79	5.51
	Median	3	2	3	4
	Standard Deviation	1.3	1.3	1.33	3.26
	Minimum Value	1	1	1	1
	Maximum Value	6	7	7	14

## DISCUSSION

**Figure 3:**
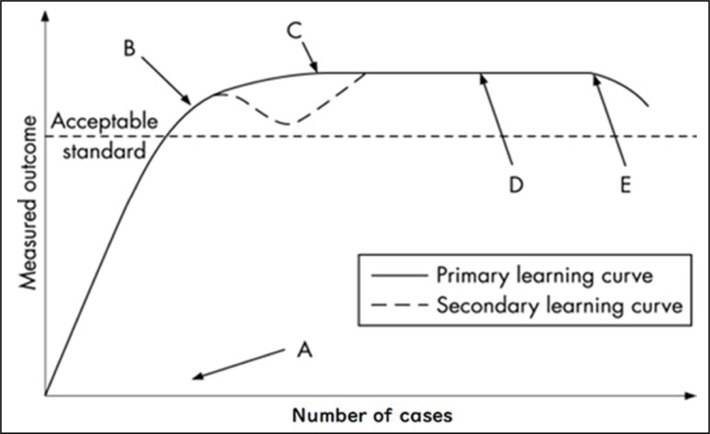
Hopper learning curve. A: Training start, B: Ability to perform the procedure competently and independently, C: Gain of professional experience doesn't change the final result, D: Plateau, E: Drop in performance with advancing age.

Clubfoot is a public health matter, affecting 200,000 children every year in the world. The consequences when clubfoot is not treated can be devastating and can cause a high social and financial impact on patients, families and in the health care system.^4^ Ponseti method provides good results, is low cost and highly reproducible when not modified.^14^ However, close attention should be paid to details. Incorrect casting can lead to complication as complex clubfoot with requires even more specific and delicate treatment. Learning curve for medical procedures involving surgeries was designed for the United Kingdom’s Health Ministry by Hoper and it has 4 main phases. First phase represents the beginning of training; in the second phase the curve ascends, and the gradient of this ascent indicates how quicky individuals’ performance improves and may be a stepwise ascent as individuals learn and master stages of complex procedure. Improvements in performance tend to be most rapid at first and then tail off, as the degree of improvement attained with each case reduces as techique is refined. In the third phase, assuming an adequate aptitude, a point is reached when the procedure can be performed both independently and competently. Additional experience improves outcomes by very small amounts, until a plateau is reached. In the fourth phase, with advancing age, manual dexterity, eyesight, memory and cognition may deteriorate, outweighing any advantage from long experience, leading to a fall in the level of performance. An alternative curve has been also described, which exhibits temporary performance deterioration after technical competence has been achieved and the probable reasons are technical adaptations or over confidence resulting in lapses in technique or judgement.^15^ (Figure 3)

Many orthopedic procedures such as resurfacing hip arthroplasties^16, 17^ and the treatment of hip dysplasia^18^ had described their learning curve. The learning curves show that practitioners improve their results and decrease complications as the experience increases. The purpose of our study was to demonstrate the orthopedic surgeon learning curve in the Ponseti method to treat idiopathic clubfoot considering the number of casts, treatment time and the correction progression according to the Pirani Score. We found that in the beginning of practice both practitioners obtained correction of the deformities in a similar way regarding number of casts, but as the mentor became more experienced over time the number of casts needed to obtain correction of the deformities decreased. The mentor’s learning curve demonstrated that the number of casts to correct the feet in the second interval (2005 to 2010) was fewer than in the first interval (2000 to 2005), but was similar to the third interval (2010 to 2015). It is possible to compare this curve to the drawing of Hoper learning curve.^19^ The first interval as the coordinate A, in the beginning of training the curve raises demonstrating the gradually performance improvement as the practitioner learns and masters treatment techniques. The second interval as coordinate B in Hoper’s learning curve drawing, when the practitioner is adequately qualified and can perform the procedure independently and competently. In the learning of the Ponseti Method, the alternative curve between coordinates B and C that exhibits temporary deterioration in performance after technical competence has been achieved may represent the period in which the practitioner has already reached technical competence and starts to make adaptations to the Ponseti method, in addition to mixed and more complex cases. Mentors third and last interval is represented by coordinate C, where additional experience improves results in very small amounts, suggesting a plateau on the learning curve (Figures 1 and 3). We can also infer that the learning curve in the Ponseti method is a tool that helps the training orthopedic surgeon self-evaluation.

The mentor’s evolution showed that the number of casts necessary to obtain correction decreased as the gain of experience increased, especially in the second interval five years after the beginning of practice. This evolution characterizes the learning curve in the Ponseti method to treat idiopathic clubfoot.

Both centers treated more than 50 clubfeet. The Ponseti International Association (PIA) guidelines recommend as able to treat clubfoot patients the professional who has treated at least 50 feet.^20^

Consequences when not treating a congenital clubfoot can be devastating. In ambulatory children, the deformity causes the child to walk on the lateral border of their foot. The social, emotional and financial consequences of non treated clubfoot are felt for a lifetime.^20,21^

The practice of many orthopedic procedures has already shown that there is an improvement of results and reduction of complications with the gain of experience.^16–18^ Considering the number of casts per patient to obtain correction of deformities, both the mentor (average of 3 casts per patient) and the trainee (average of 5 casts per patient) managed to correct the deformities of clubfoot within the number of casts changes suggested by Dr Ponseti.^3,22^

The Pirani Score measure clubfoot severity visually, dynamic and tactile and assists the orthopedic surgeon in the learning curve of the Ponseti method.^7^

Indication for tenotomy is a corrected foot concerning forefoot alignment and an abduction of 70° degrees. The equinus correction through the subtalar joint is maximal, lacking only final degrees of ankle dorsiflexion. The authors had performed tenotomy in 98% of cases. No foot needed extensive surgery such as posterior or posteromedial release.

The most important decrease of the Pirani Score for all patients occurs from the first to the second cast and it’s associated with the forefoot. Hindfoot deformities are the last to be corrected, and the equinus usually is obtained only with the tenotomy of the Achilles tendon.

There was no correlation between the severity of the Pirani score and time until tenotomy, meaning that the greater severity of the foot is not an indication that patient will need more casts until correction.^23,24^ This is a relevant fact as the progression of correction depends more on how the foot will answer to treatment than to practitioner’s expertise.

Patient’s age, which often drives families away from less invasive treatment and may be a demotivating factor for the orthopedic surgeon to start the treatment, was not related to the number of casts (patients age from 14 to 180 days old). This finding was also observed by an European study comparing the success of the Ponseti method in patients younger and older than 6 months old.^25^ An American study demonstrated that there is no urgency in start treatment in clubfoot newborn patients.^15^

Since 2016, PIA Brasil (Ponseti International Association affiliated), team formed by a group of pediatric orthopedic surgeons concerned with the correct diffusion and application of the Ponseti method, joined a partnership with Rotary International and with a global grant started a national training program. This was an educational program that has already trained 50 orthopedic practitioners of the Ponseti method in the mentorship model with the aim of improving the technique and creating a net of reference centers throughout the country. Differentials of the mentoring model include the close mentor/ trainee relationship, hands-on practice, contact with patients at various stages of treatment and case discussion with experienced mentors. This may collaborate for acceleration of young practitioners learning curves. This training program has already been replicated in other countries in Latin America.

We suggest that administration support, parent’s groups support, and mentoring model training are relevant in the learning curve of the Ponseti method.

## CONCLUSION

The experience of the orthopedic surgeon results in a shorter treatment time and fewer cast changes, when three 5-year interval times are analyzed, regardless foot’s side or severity and patient’s age or gender. The Pirani Score also followed the same pattern, characterizing Ponseti method learning curve.
